# From ELSA to responsible research and *Promisomics*

**DOI:** 10.1186/2195-7819-9-3

**Published:** 2013-05-15

**Authors:** Ruth Chadwick, Hub Zwart

**Affiliations:** Cardiff University – School of English, Communication & Philosophy, 6 Museum, Place Cardiff, F10 3AX UK; Department of Philosophy and Science Studies, Radboud University Nijmegen, Faculty of Science, Institute for Science, Innovation and Society (ISIS), P.O. Box 9010, 6500 GL Nijmegen, The Netherlands; Centre for Society and the Life Sciences (CSG), P.O. Box 9010, 6500 GL Nijmegen, The Netherlands

## Scope and objectives of the new journal

It is with great pleasure that we hereby formally launch *Life Sciences, Society and Policy* (LSSP) as a peer-reviewed, open access journal devoted to fostering scholarly research into the ethical, legal and social dimensions of emerging life sciences. LSSP provides an academic forum for engaged scholarship, interdisciplinary research, critical reflection and informed discussion to promote responsible life sciences research and sustainable development. Besides academic reflections, deliberations and debates, LSSP provides insights, tools and recommendations for civil society, policy, industry and education. Its aim is to analyse, assess and improve the interrelatedness of emerging life sciences, society and policy.

The journal covers a broad area of research and reflection, probing and questioning the meaning of new forms of knowledge concerning human beings, animals, plants and the environment. These issues are addressed from a truly international and global perspective and LSSP welcomes submissions from all countries around the globe on a broad range of subjects, ranging from normative or theoretical reflections up to quantitative or qualitative research and case studies and is keen on publishing themed sections as well.

Since the emergence of genomics, notably its highly visible flagship endeavour: the *Human Genome Project* (HGP), a whole series of –omics research fields have emerged, such as proteomics, metabolomics, transcriptomics, cognomics, and connectomics, as well as other large-scale post-genomics endeavours such as systems biology, synthetic biology and epigenetics. As a rule, these initiatives tend to give rise to quite substantial promises and expectations for society. Yet, this type of promissory discourse (‘promisomics’) runs the risk of becoming inflated, and may entail loss of credibility for science. In our view, responsible promise management should be part of responsible research, the outcome of an on-going dialogue between science and society. Thus, the articulation of feasible promises and expectations, as a collective, trans-disciplinary effort, is an important objective of our journal.

## A short history of LSSP

In 2005, *Genomics, Society and Policy* (GSP), the predecessor of LSSP, was launched as a peer-reviewed freely accessible on-line journal, publishing articles and reviews on the societal dimensions of emerging life sciences, notably genomics. GSP was developed within the *ESRC Genomics Network* (EGN) in the UK, and launched in 2005 as a co-production with the *Centre for Society and Genomics* (CSG) in the Netherlands. From the beginning, CSG has functioned as an important outlet for the ELSA genomics/life sciences community that has now evolved worldwide. GSP published 8 issues (2005–2012) which are now available as an integrated part of the LSSP archive.

From 2013 onwards, Springer Science & Business Media will publish the journal as an Open Access publication. As our scope has broadened and now includes post-genomics areas such as personalised medicine, systems biology and synthetic biology as well, we decided to change the name of the journal into LIFE SCIENCES, SOCIETY AND POLICY.

## The ELSA life sciences community

In order to adequately address the societal dimensions of emerging life sciences in all their complexity, a trans-disciplinary approach is called for. Therefore, LSSP gives the floor to a broad spectrum of disciplines and approaches, such as philosophy of science, bioethics, science and technology studies, science communication, technology assessment and others. Usually, this field is referred to as ELSI or ELSA research.

The acronym ELSI (in the U.S.) or ELSA (in Europe) refers to research activities that anticipate and address ethical, legal and social *implications* (ELSI) or *aspects* (ELSA) of emerging life sciences, notably genomics. ELSI was conceived in 1988 when James Watson, at the press conference announcing his appointment as director of the Human Genome Project (HGP), suddenly and somewhat unexpectedly declared that the ethical and social implications of genomics warranted a special effort and should be directly funded by NIH (Cook-Deegan [1994/1995]). Over the years, various ELSI or ELSA programs have been developed, in Canada,^a^ Europe and the Far East. Table 1 provides a historical overview, to which several new initiatives could be added.

**Table 1:** **Overview of ELSI/A programs**

Country	Acronym	Program	Funding agency	Year
U.S.A.	ELSI	Ethical, Legal and Social Implications	NIH/NHGRI	1990
Canada	GE^3^LS	Genomics-related Ethical, Environmental, Economic, Legal and Social Aspects	Genome Canada	2000
South-Korea	ELSI	Ethical, Legal and Social Implications	Government of South-Korea	2001
United Kingdom	EGN	ESRC Genomics Network (Cesagen, Innogen, Egenis, Genomics Forum)	ESRC	2002
Netherlands	CSG, MCG	Centre for Society and Genomics (now: Life Sciences); Societal Component of Genomics Research	Netherlands Genomics Initiative	2002
Norway	ELSA	ELSA Program	Research Council of Norway	2002
Germany, Austria, Finland	ELSAGEN	Transnational Research Programme	GEN-AU, FFG, DFG, Academy of Finland	2008

In Kant [1798/1971], published his book “The conflict of the faculties” (*Die Streit der Fakultäten*), arguing that philosophy should critically reflect on the research that is being conducted within the other three faculties that existed in those days, namely Theology, Medicine and Law - the 18th century equivalents of what we nowadays refer to as humanities, natural science and social science. Following Kant, philosophers should critically reflect on how these other disciplines address issues such as informed consent and the use of research animals (in Medicine), individual autonomy (in the case of Theology) and justice (in the case of Law). This Kantian approach experienced a dramatic come-back after the Second World War when traumatic experiences such as the Nuremberg Trials and nuclear bombing raised grave societal concerns. In an era of next generation sequencing and synthetic cells, these concerns have far from evaporated, while the field of Science Studies addressing them has significantly broadened, so that it now involves a broad spectrum of disciplines besides philosophy proper. In Europe, these issues are currently addressed under the heading of Responsible Research and Innovation (RRI), calling upon research consortia to make these reflections and deliberations an integrated part of their work.

We hope that this journal will allow us to share insights and experiences and consolidate our field and develop our expertise.

## Endnote

^a^The Canadian acronym (GE^3^LS) added the G (for genomics) but dropped the postfix A (or I).

## References

[CR1] Cook-Deegan R (1995). The gene wars: science, politics and the human genome.

[CR2] Kant I, Weischedel W (1971). Der Streit der Fakultäten. Sämtliche Werke in zehn Bänden IX.

